# Mapping and systematic appraisal of umbrella reviews in epidemiological research: a protocol for a meta-epidemiological study

**DOI:** 10.1186/s13643-023-02265-7

**Published:** 2023-07-14

**Authors:** Lazaros Belbasis, Robin D Brooker, Emmanuel Zavalis, Angelo Maria Pezzullo, Cathrine Axfors, John PA Ioannidis

**Affiliations:** 1 Meta-Research Innovation Center Berlin, QUEST Center, Berlin Institute of Health, Charité–Universitätsmedizin Berlin, Berlin, Germany; 2 Clinical Trials and Epidemiological Studies Unit, Nuffield Department of Population Health, University of Oxford, Oxford, UK; 3 Department of Sociology, University of Essex, Colchester, UK; 4 Meta-Research Innovation Center at Stanford, Stanford University, Stanford, USA; 5 Section of Hygiene, Department of Life Sciences and Public Health, Università Cattolica del Sacro Cuore, Rome, Italy; 6 Department of Women’s and Children’s Health, Uppsala University, Uppsala, Sweden; 7 Department of Medicine, Stanford University School of Medicine, Stanford, CA USA; 8 Department of Epidemiology and Population Health, Stanford University School of Medicine, Stanford, CA USA; 9 Department of Biomedical Data Science, Stanford University School of Medicine, Stanford, CA USA; 10 Department of Statistics, Stanford University School of Humanities and Sciences, Stanford, CA USA

**Keywords:** Critical appraisal, Meta-analysis, Meta-research, Systematic review, Umbrella review

## Abstract

**Introduction:**

Umbrella review is one of the terms used to describe an overview of systematic reviews. During the last years, a rapid increase in the number of umbrella reviews on epidemiological studies has been observed, but there is no systematic assessment of their methodological and reporting characteristics. Our study aims to fill this gap by performing a systematic mapping of umbrella reviews in epidemiological research.

**Methods:**

We will perform a meta-epidemiological study including a systematic review in MEDLINE and EMBASE to identify all the umbrella reviews that focused on systematic reviews of epidemiological studies and were published from inception until December 31, 2022. We will consider eligible any research article which was designed as an umbrella review and summarized systematic reviews and meta-analyses of epidemiological studies. From each eligible article, we will extract information about the research topic, the methodological characteristics, and the reporting characteristics. We will examine whether the umbrella reviews assessed the strength of the available evidence and the rigor of the included systematic reviews. We will also examine whether these characteristics change across time.

**Discussion:**

Our study will systematically appraise the methodological and reporting characteristics of published umbrella reviews in epidemiological literature. The findings of our study can be used to improve the design and conduct of future umbrella reviews, to derive a standardized set of reporting and methodological guidelines for umbrella reviews, and to allow further meta-epidemiological work.

**Systematic review registration:**

osf.io/sxzc6

## Introduction

Systematic reviews and meta-analyses are important components in the chain of scientific information [1]. They constitute key tools for evidence-based medicine and an important research design for appraising evidence and guiding medical practice and health policy [1, 2]. During the last decades, there is a very rapid increase in the number of published systematic reviews and meta-analyses, and often there are multiple overlapping or complementary systematic reviews and meta-analyses for numerous research questions [1].

In this research landscape, it is often very important to examine the evidence not only on a single question, but on multiple questions on a given topic. By summarizing the evidence from multiple systematic reviews and meta-analyses, researchers achieve a thorough integration of evidence and provide a bird’s eye view on a broad topic [2, 3]. Overviews of reviews, which compile data from multiple systematic reviews, emerged to deal with the growing volume of published systematic reviews [4]. Alternative terms have also been used to refer to overviews, including reviews of systematic reviews, and umbrella reviews [5].

Although the term “umbrella review” appeared more than a decade ago, its use became popular recently [2]. Indeed, based on our preliminary literature search, more than 80% of the research articles using the term “umbrella review” were published during the last 5 years, and about 60% of them focused on epidemiological evidence. Umbrella reviews have been previously described as a systematic collection and assessment of multiple systematic reviews and meta-analyses on a specific research topic [2, 6], a definition that is equivalent to the one for overviews of systematic reviews.

Previous meta-epidemiological work has been done to assess the methodological and reporting characteristics and the quality of overviews of systematic reviews [7–12]. However, these studies considered overviews published before 2017, when the use of the “umbrella review” term was not very prevalent, and they focused on overviews of systematic reviews on clinical evidence. Also, a couple of studies performed a bibliometric analysis of overviews including articles published more recently, but they examined only publication and co-authorship patterns without assessment of methodological and reporting characteristics [13, 14]. The results from these meta-epidemiological studies have been used to produce the PRIOR statement [5].

However, systematic reviews of observational studies have different biases and difficulties to consider, and findings from existing meta-research, which focuses on overviews of systematic reviews on randomized trials, might not be generalizable. Until now, there is only one published overview of umbrella reviews on meta-analyses of observational studies, which focused exclusively on the approaches used to grade the epidemiological associations [15]. In the present protocol, we describe a meta-epidemiological study which aims (a) to map the use of the umbrella review methodology in the epidemiological literature and (b) to assess the methodological and reporting characteristics of umbrella reviews on epidemiological evidence.

## Methods

### Literature search

The present research protocol describes a meta-epidemiological study, which is based on a systematic literature review. We will search MEDLINE and EMBASE (using Ovid) to identify umbrella reviews that have been published from inception to December 31, 2022. Our search algorithm is based on published recommendations for the retrieval of overviews of systematic reviews and is presented in Table 1 [16]. Our protocol is registered in Open Science Forum (osf.io/sxzc6).

**Table 1:** Search algorithm in MEDLINE and EMBASE through Ovid

#	Search keywords	Results
1	overview$.ti	108,964
2	review.ti	1,453,974
3	synthesis.ti	782,922
4	summary.ti	42,580
5	cochrane.ti	7783
6	analysis.ti	2,519,554
7	1 or 2 or 3 or 4 or 5 or 6	4,659,119
8	reviews.ti	27,163
9	meta-analyses.ti	10,716
10	articles.ti	15,515
11	umbrella.ti	4259
12	8 or 9 or 10 or 11	54,662
13	7 and 12	17,585
14	"umbrella review".ab	2117
15	meta-review.ti,ab	707
16	metareview.ti,ab	57
17	15 or 16	753
18	13 or 14 or 17	18,415
19	limit 18 to english language	17,635
20	limit 19 to humans	14,377
21	limit 20 to yr = "1990—2022"	13,632

### Eligibility criteria

We will include all the research articles that performed a systematic collection and assessment of multiple systematic reviews and meta-analyses of epidemiological studies (e.g., risk factors, individual predictors or prognostic factors of a disease or quantitative trait, and prevalence and/or incidence of a disease). Eligible articles could use different terms to describe their study design, such as umbrella review, or overview. However, we will not include research articles that systematically collected and assessed systematic reviews and/or meta-analyses of clinical trials, because extensive meta-epidemiological work has been done on this field [7–12]. We will include only articles published in English. We will exclude preprints, commentaries, narrative reviews, methodological papers, conference abstracts, and research protocols.

Two researchers (LB, RDB, EZ, AMP, CA) will independently screen all resulting articles from the literature search to assess their eligibility, and disagreements will be resolved after consulting a third researcher (JPAI). The screening of articles will be performed in three phases (i.e., title, abstract, and full-text screening). In each phase, the reasons for exclusion will be recorded and a summary of these reasons will be presented.

### Data extraction

To facilitate the data extraction process, we will consider the PRIOR checklist [5], and two researchers (LB, JPAI) will construct a data extraction form. Two researchers (LB, RDB, EZ, AMP, CA) will independently extract the data from the eligible studies, and disagreements will be resolved after consulting a third researcher (JPAI). In the data extraction process, we will consider both the main publication and the supplementary material of the eligible articles. A summary of the extracted items is presented in Table 2.

**Table 2:** Items extracted from the eligible umbrella reviews

Category	Extracted items
*Metadata*	
General characteristics	First author
	Date of publication
	Journal of publication
*Introduction*	
Research scope	Research topic
	Study designs of interest
*Methods*	
Literature search	Bibliographic databases
	Date of literature search
	Handling of SRMAs on overlapping research question
	Quantification of the overlap in the primary studies across overlapping SRMAs
	Update of eligible SRMAs
	Search for Mendelian randomization studies^a^
Statistical methods	Narrative discussion of SRMAs without statistical analysis
	Presence of meta-analysis
	Meta-analytic model applied
	Statistical significance threshold
	Between-study heterogeneity
	Tests for bias
	95% prediction intervals
	Sensitivity analyses
Risk of bias	Qualitative assessment of primary studies
	Qualitative assessment of eligible SRMAs
	Assessment of the strength of the evidence
Open science principles	Protocol pre-registration
	Data sharing agreement
*Results*	
	Number of eligible SRMAs
	Number of meta-analyses performed
	Number of statistically significant associations
	Reporting of the eligible SRMAs
	Flow chart for study selection
	Reporting of results in tabular and/or visual format
*Discussion*	
Existing literature	Discussing previous umbrella reviews^a^
	Discussing Mendelian randomization studies^a^

*SRMAs* systematic reviews and/or meta-analyses

^a^The annotated items are not included in the PRIOR checklist

From each eligible umbrella review, we will extract information about the first author, the year and journal of publication, and the research topic of interest. We will categorize the scope of the eligible umbrella reviews into (a) risk factors for a disease, medical condition or quantitative trait, (b) individual predictors or prognostic factors or multivariable models for a disease, medical condition or health-related outcome, (c) incidence or prevalence of a disease or medical condition, and (e) other. We will examine whether the eligible umbrella reviews followed an environment-wide approach (i.e., considering the association of multiple risk factors, individual predictors or prognostic factors with a single disease or health-related outcome), a phenome-wide approach (i.e., considering the association of a single risk factor, individual predictor or prognostic factor with multiple diseases or health-related outcomes) or a narrow approach (i.e., considering a small number of risk factors, individual predictors or prognostic factors for a small number of diseases or health-related outcomes).

To examine the literature search process of the eligible umbrella reviews, we will extract the bibliographic databases that were searched. For each eligible umbrella review, we will take note of the date when the literature search ended and the date when the umbrella review was made available online in a scientific journal. Moreover, we will note the rules the authors applied in the case of systematic reviews and/or meta-analyses examining overlapping research questions, and whether the authors quantified the overlap in the primary studies among the overlapping systematic reviews or meta-analyses as previously suggested [10]. Also, we will record if the umbrella reviews updated the eligible systematic reviews and/or meta-analyses by searching for new primary studies. We will also examine the adherence of the eligible systematic reviews to the principles of Open Science, by recording whether there was a protocol pre-registration available and a data sharing statement.

To assess the statistical analysis of the eligible umbrella reviews, we will note whether the authors narratively described the results of already published systematic reviews and meta-analyses, and/or whether they performed additional statistical analyses. Specifically, we will examine whether the authors simply used the previously published summary results, or they reran the meta-analyses. In case that they reran the meta-analyses, we will examine if they reported the meta-analytical model applied, quantified between-study heterogeneity, performed tests for hints of bias (e.g., small-study effects and excess significance bias), calculated 95% prediction intervals, and performed sensitivity analyses for all or a part of the meta-analyses. We will also examine if the authors graded the strength of the evidence from each meta-analysis. If yes, we will record the criteria they applied. We will also examine if the researchers extracted the qualitative assessment of the primary studies as presented by the eligible systematic reviews and if the researchers performed a qualitative assessment of the included systematic reviews by applying a standardized assessment tool, such as AMSTAR or variants thereof [17].

Overviews of systematic reviews often do not report previously published overviews on the same research question [12]. In our study, we will explore if this is the case for umbrella reviews on epidemiological evidence. When multiple umbrella reviews on the same research question are available, we will examine whether an umbrella review mentions the previously published umbrella review(s) and whether overlapping umbrella reviews have the same conclusions. If not, we will explore potential reasons including differences in the search strategy, inclusion criteria, statistical analysis, and grading criteria.

To examine the reporting of the eligible umbrella reviews, we will extract the number of eligible systematic reviews and/or meta-analyses included in each umbrella review, and the total number of meta-analyses performed. We will also examine whether they reported all the references of the eligible systematic reviews and meta-analyses and whether a flow chart showing the study selection process is available. We will record the format of the presentation of the results (tabular and/or visual).

Among the umbrella reviews of epidemiological associations, we will also examine whether the authors systematically collected Mendelian randomization studies or if they considered the results of Mendelian randomization studies in their discussion. Based on this, we will categorize umbrella reviews into articles systematically collecting Mendelian randomization studies, articles narratively discussing findings from Mendelian randomization studies without a prior systematic search, and articles that did not mention Mendelian randomization studies.

### Statistical analysis

We will present descriptive statistics for the methodological and reporting characteristics we captured by calculating the median and the interquartile range for continuous variables, and counts and frequencies for binary and categorical variables. We will assess whether the publication patterns and the methodological and reporting characteristics of umbrella reviews change over time using exact tests for binary variables and analysis of variance for continuous variables. We hypothesize that over time, there will be larger number of eligible umbrella reviews of epidemiological studies published per year, a large number of included systematic reviews per umbrella review, a larger proportion of umbrella reviews that are pre-registered, a larger number of umbrella reviews that have data sharing statements, and a larger proportion of umbrella reviews that formally assess overlap between systematic reviews with quantitative methods.

We will set the level of statistical significance at *P* value < 0.005 with *P* values between 0.05 and 0.005 being considered suggestive. Statistical analysis will be performed using R version 4.2.2.

## Discussion

There is an increasing number of published umbrella reviews on various research topics. For this reason, it is important to track and appraise umbrella reviews by examining their methodological and reporting characteristics. To address this need, our study will provide a systematic and critical mapping of the published umbrella reviews in epidemiological literature. The main output of our study will be an overview of the subject matter, methodological and reporting landscape of published umbrella reviews.

Multiple meta-epidemiological studies made substantial contribution to the methodology of overview of systematic reviews during the last decade. It has been shown that overviews often neglect the up-to-dateness of the eligible systematic reviews [11]. Also, the extent of overlap among overlapping systematic reviews is often neglected and even when the presence of overlap is reported, it is not adequately quantified [12]. Moreover, the reporting and methodological quality of eligible systematic reviews often are not assessed, and reasons for discordance among overlapping systematic reviews are usually not examined [9].

The expected output of our meta-epidemiological study is a catalogue and a detailed methodological and reporting assessment of the available umbrella reviews on epidemiological evidence. Eventually, we expect that our findings can be used to improve the design and conduct of future umbrella reviews. They could also serve as the basis for the development of methodological guidelines and recommendations. Finally, the database of the collected umbrella reviews may serve as the basis for further meta-epidemiological research in the future. Possibilities for such research efforts include (but are not limited to) the in-depth appraisal of the evidence procured by umbrella reviews and its comparison against different types of evidence syntheses; assessments of the landscape of redundant or overlapping meta-analyses; and the comparison of different types of study designs in addressing the same question (e.g., prospective versus retrospective studies).

## Data Availability

The present article is a research protocol. Data will be made publicly available after the publication of the relevant research article.
